# Myocardial haemodynamic responses to dobutamine stress compared to physiological exercise during cardiac magnetic resonance imaging

**DOI:** 10.1186/1532-429X-15-S1-P16

**Published:** 2013-01-30

**Authors:** Kaleab N Asrress, Andreas Schuster, Nafees F Ali, Rupert Williams, Shelby Kutty, Tim Lockie, Mehreen Yousuff, Kalpa De Silva, David A Danford, Philipp Beerbaum, Michael Marber, Sven Plein, Eike Nagel, Simon Redwood

**Affiliations:** 1 Cardiovascular Sciences, King's College London, London, UK; 2 Division of Imaging Sciences and Biomedical Engineering, King's College London, London, UK; 3 Multidisciplinary Cardiovascular Research Centre, University of Leeds, Leeds, UK; 4 Joint Division of Pediatric Cardiology, University of Nebraska/Creighton University, Omaha, NE, USA; 5 Department of Cardiology and Pneumology and Heart Research Center, Georg-August- University, Göttingen, Germany

## Background

Pharmacological methods remain the most common methods of inducing myocardial stress, and remain the gold standard for diagnostic purposes in the MR-environment. MR compatible ergometers now permit CMR to be performed during exercise stress, allowing physiological responses to exercise to be studied. Inotropic agents such as dobutamine are widely used in low-output states such as cardiogenic shock. However, this has consistently been shown to worsen patient outcomes. Though the effects of dobutamine are thought to mimic that of exercise stress, exercise is known to have a beneficial effect on patient outcomes.

The aim of this study was to investigate the effects of these two stress methods on myocardial haemodynamics and deformation to gain insight as to factors that may contribute to their differing impacts on prognosis.

## Methods

Two groups were studied consisting of 10 healthy volunteers without known cardiovascular disease. A standard series of steady state free precession (SSFP) sequences were acquired at rest and during intermediate dose dobutamine infusion (20 µcg/kg^-1^/min^-1^) at 1.5-Tesla. Exercise studies were performed on a separate group of volunteers using a standardised incremental supine bicycle ergometer protocol (Lode, Groningen Netherlands) at 3.0-Tesla. Myocardial deformation parameters were performed using custom software (Feature Tracking, Tomtec, Germany) to analyse standard SSFP cine images from the mid-short axis slice. Haemodynamic parameters were recorded throughout the studies.

## Results

Table 1 shows haemodynamic data, left ventricular volumes and strain parameters at rest, during intermediate dose dobutamine infusion and exercise stress. Resting parameters were not significantly different between the two studies. Dobutamine and exercise increased heart rate and stroke volume by similar degrees. They both increased cardiac index, stroke volume and ejection fraction significantly, but exercise achieved this while maintaining end diastolic volumes. Dobutamine and exercise both increased contractility by increasing strain and reducing the time to achieve peak strain; dobutamine significantly greater than exercise. Despite this there is a greater augmentation of blood pressure with exercise.

**Table 1:** The effects of intermediate dose dobutamine compared to exercise stress on left ventricular haemodynamics and strain parameters.

Parameter	Stressor				Significance			
	Rest 1	Rest 2	Exercise	Dobutamine	Rest 1 vs rest 2	Rest vs exercise	Rest vs dobutamine	Exercise vs dobutamine

Heart Rate (bpm)	70.2±13.7	68.6±11.9	108±10	116±11	NS	<0.05	<0.05	NS
Mean BP (mmHg)	89.4±6.4	91.5±10.2	124±19	103±11	NS	<0.05	<0.05	<0.05

CI (l/min/m^2^)	2.7±0.6	3.0±0.6	4.5±1.1	5.7±0.8	NS	<0.05	<0.05	<0.05
EDV (ml/m^2^)	73.2±15.9	76.9±12.5	72.0±17.2	64.5±11.5	NS	NS	<0.05	<0.05
ESV (ml/m^2^)	34.5±9.7	33.4±7.5	25.9±9.4	15.2±5.0	NS	<0.05	<0.05	<0.05
SV (ml/m^2^)	38.6±7.7	43.5±6.5	46.1±8.5	49.3±8.0	NS	<0.05	<0.05	NS
EF (%)	53.3±6.3	56.9±4.4	65.0±5.7	76.9±4.6	NS	<0.05	<0.05	<0.05

Err (%)	21.3±9.7	19.6±9.2	33.0±16.1	42.3±18.5	NS	NS	<0.05	NS
Err.tps (ms)	294±62	345±90	267±52	187±34	NS	<0.05	<0.05	<0.05
Ecc (%)	22.6±2.9	24.1±2.5	30.6±2.9	39.2±8.3	NS	<0.05	<0.05	<0.05
Ecc.tps (ms)	305±64	338±57	288±63	179±20	NS	<0.05	<0.05	<0.05

Rest 1= rest prior to exercise study; Rest 2=rest prior to dobutamine study. Left ventricular volumes are indexed for body surface area and results are expressed as mean ± standard deviation. Paired t-test was used to test significance, set at a level of <0.05. CI= cardiac index, EDV=end diastolic volume, ESV=end systolic volume, SV=stroke volume, EF=ejection fraction, Err=short axis radial strain, Err.tps=radial time to peak strain, Ecc=circumferential strain, Ecc.tps=circumferential time to peak strain.

## Conclusions

Compared to exercise stress, dobutamine has similar effects on stroke volume and cardiac output but greater impact on contractility. However this is at the expense of diastolic physiology and relaxation, both important factors for myocardial perfusion. Therefore to mimic the effects of exercise, inotropes should ideally possess an ability to increase contractility without adversely affecting diastolic relaxation and filling. CMR provides a novel tool with which these processes can be studied in humans non-invasively.

## Funding

KA receives grant support from the British Heart Foundation (BHF) (FS/11/43/28760) and the National Institute for Health Research (NIHR) via the comprehensive Biomedical Research Centre (BRC) award to Guy's and St Thomas' NHS Foundation Trust. AS receives grant support from the BHF (RE/08/003 and FS/10/029/28253) and the BRC. RW receives grant support from the BHF. SK receives grant support from the American College of Cardiology Foundation, the Edna Ittner Pediatric Foundation, and the Children's Hospital and Medical Center Foundation. EN receives grant support from BHF, the Wellcome Trust and Engineering and Physical Sciences Research Council and the BRC. SP receives grant support from the BHF.

